# Emotion regulation and peripheral psychophysiological correlates in the management of induced pain: A systematic review

**DOI:** 10.1371/journal.pone.0253509

**Published:** 2021-06-29

**Authors:** Irene Jaén, Amanda Díaz-García, M. Carmen Pastor, Azucena García-Palacios

**Affiliations:** 1 Basic Psychology, Clinical Psychology and Psychobiology, Universitat Jaume I, Castello de la Plana, Spain; 2 Psychology and Sociology, Universidad de Zaragoza, Zaragoza, Spain; University of Essex, UNITED KINGDOM

## Abstract

Cognitive reappraisal and acceptance strategies have been shown to be effective in reducing pain experience and increasing pain tolerance. However, no systematic reviews have focused on the relationship between the use of these two strategies and peripheral physiological correlates when pain is experimentally induced. This systematic review aims to summarize the existing literature that explores the relationship between emotion regulation strategies (i.e., cognitive reappraisal and acceptance) and peripheral correlates of the autonomic nervous system and facial electromyography, such as affect-modulated responses and corrugator activity, on laboratory tasks where pain is induced. The systematic review identifies nine experimental studies that meet our inclusion criteria, none of which compare these strategies. Although cognitive reappraisal and acceptance strategies appear to be associated with decreased psychological responses, mixed results were found for the effects of the use of both strategies on all the physiological correlates. These inconsistencies between the studies might be explained by the high methodological heterogeneity in the task designs, as well as a lack of consistency between the instructions used in the different studies for cognitive reappraisal, acceptance, and the control conditions.

## Introduction

The conceptualization of pain has undergone a great evolution over the years. The first definitions highlighted a direct correspondence between the damage and the experienced pain. However, the understanding of pain has evolved considerably from its inception, and it is currently considered a multifaceted phenomenon composed of both sensory-discriminative and motivational-affective dimensions [1,2]. Thus, pain is defined as “an unpleasant sensory and emotional experience associated with, or resembling that associated with, actual or potential tissue damage or described in terms of such damage” [3]. This definition recognizes the association between tissue injury and pain, as well as the sensory and emotional dimensions of the experience [4].

A large number of studies have shown the relationship between pain and emotion, proposing emotions as determinants and consequences of the subjective pain experience [5,6]. Thus, pain can be modulated by emotions, with numerous studies showing that inducing negative affect is related to elevated self-reported pain ratings and lower pain tolerance, whereas inducing positive affect is related to less self-reported pain and higher pain tolerance [7–10]. In this line, recent research has proposed that emotion regulation (ER) can be an important factor in the development and management of pain [11].

ER is defined as the ability to modify emotional responses in behavioral, experiential, or physiological domains to achieve one’s goals [12,13]. Many studies have shown the efficacy of ER strategies for modulating negative emotions such as anger, fear, or sadness [14,15]. Specifically, acceptance, suppression, avoidance rumination, problem-solving, and cognitive reappraisal were studied and synthesized in a large meta-analysis conducted by Aldao et al. [16], showing that rumination, avoidance, and suppression can be considered maladaptive ER strategies, whereas problem-solving, reappraisal, and acceptance can be classified as adaptive strategies. These latter two strategies (i.e., reappraisal and acceptance) have been widely studied in relation to pain. They are core elements of first-line evidence-based treatments for pain management, such as Cognitive Behavioral Therapy (CBT) [17] and Acceptance and Commitment Therapy (ACT) [18]. Cognitive reappraisal is an antecedent-focused strategy that involves changing the meaning and emotional valence of a stimulus to change its emotional impact [12,19]. For example, in pain research, cognitive reappraisal has been taught to participants using positive self-statements that emphasize the individual’s ability to tolerate pain (e.g., “I can stand this”) or underestimate the pain (“It’s not that bad”) [20], reinterpreting sensory experiences (e.g., imagining thermal stimulation as a blanket on a cold day) [21], or changing the meaning of the stimuli to modify the emotional impact of negative stimuli (e.g., “This is good for your health”) [22]. Acceptance is a response-focused strategy that does not aim to change the meaning of the stimulus, but rather changes the way the person relates to his/her thoughts and feelings [23]. Hayes et al. [18] refers to acceptance as the willingness to remain in contact with and actively experience particular private experiences that are accompanied by functional behaviors. McCracken, Vowles, & Eccleston [24] argued that acceptance of pain consists of two components. The first component is concerned with the individual engaging in positive and functional activities in a normal way in spite of experiencing pain. The second component has to do with the recognition that avoiding or controlling pain is ineffective. Thus, in the context of pain, studies could instruct participants to notice their thoughts and feelings but continue with the task in order to achieve their goal [25]. Moreover, some studies model their instructions on broader mindfulness-based interventions and include, in addition to acceptance, other mindfulness facets such as observing and non-judging. For example, participants can be instructed to attend to their feelings and accept the experience, without judging the “goodness” or “badness” of this sensation [26].

Laboratory studies have shown that both ER strategies (i.e., reappraisal and acceptance) are effective in down-regulating negative emotions, resulting in less negative self-reports [27,28]. Moreover, these strategies are not only useful for modulating the subjective experience, but they can also produce changes in psychophysiology, including the autonomic nervous system (e.g., electrodermal activity; heart rate) and affect-modulated (e.g., startle reflex) and behavioral (e.g., corrugator) responses [29–31] when facing negative stimuli. In this regard, both reappraisal and acceptance have been shown to be effective in decreasing electrodermal activity and heart rate responses [27,32,33]. Likewise, reappraisal has also been associated with diminished defensive responses such as the startle reflex [32,34], although the literature shows a lack of agreement in the results [35–39]. Regarding behavioral responses, studies have reported that corrugator activity decreases when participants use reappraisal or acceptance strategies [28].

These two strategies have also been shown to be effective in reducing the pain experience and increasing pain tolerance, measured with subjective ratings [25,40–42]. However, the down-regulation of both negative emotions and pain experience is not always accompanied by the expected psychophysiological responses [43,44]. Mixed results have been reported in this regard, possibly due to the methodological heterogeneity across the studies. Therefore, there is a need to summarize all the studies on the relationship between the use of ER strategies (reappraisal and acceptance) and psychophysiology, in order to identify which ER instructions and other methodological factors influence this relationship. To our knowledge, only one systematic review has summarized the existing studies on the association between ER and pain [11]. However, this review does not include studies with psychophysiological measures. To the best of our knowledge, no systematic reviews have focused on studies that use experimental tasks to assess the relationship between psychophysiological activity and the use of ER strategies, specifically reappraisal and acceptance, for pain management. ER encompasses the measurement of cognitive, behavioral, and psychophysiological responses to an event or stressor [45]. Hence, psychophysiological measures can offer important advantages in the study of ER strategies, providing relevant information about changes in internal experiences that cannot be assessed with subjective measures. For this reason, this review aims to synthesize the existing literature on the relationship between emotion regulation (i.e., cognitive reappraisal and acceptance strategies) and common peripheral correlates of the autonomic nervous system and facial electromyography, such as affect-modulated responses and corrugator activity, during laboratory tasks where pain was experimentally induced.

## Methods

### Search strategy

A systematic search of the peer-reviewed literature was conducted through different databases: PubMed, Web of Science, PsycINFO and Cochrane Central Database of Controlled Clinical Trials. Additionally, Google Scholar and citations and reference lists from relevant articles were reviewed (forward and backward snowballing searches). A search for ongoing studies was performed by checking trial registries (ClinicalTrials.gov; isrctn.com). If the full-text version was not available or data were missing or unclear, the study’s authors were contacted. The terms combined to conduct the search were: “emotion regulation”, “pain”, and “psychophysiology measures”, as follows: “emotion regulat*” OR “emotional regulat*” OR “emotion dysregulation” OR “emotional dysregulation” OR “self-regulation” OR “emotional modulat*” OR “emotion modulat*” OR “emotion management” OR “emotional management” OR “emotional self-efficacy” OR “reappraisal” OR “cognitive reappraisal” OR “cognitive change” OR “acceptance” OR “affect modulation” OR “affective modulation” AND “pain” OR “pain*” OR “painful stimul*” OR “pain induction” OR “pain-induction” OR “induced pain” AND “psychophysiology” OR “psychophysiological measures” OR “electrodermal activity” OR “galvanic skin response” OR “cardiovascular” OR “cardiac defense response” OR “heart rate” OR “heart rate variability” OR “RMSSD” OR “electromyography” OR “EMG” OR “autonomic responses” OR “peripheral measures” OR “self-report*”.

The systematic review protocol was registered in the International Prospective Register of Systematic Reviews (PROSPERO) under registration number CRD42020173613.

### Eligibility criteria

Eligible studies were experimental studies that involved ER strategies, namely acceptance^1^ and cognitive reappraisal. The studies could compare these strategies to each other, to other ER strategies, or to a control condition. Acceptance has usually been included in mindfulness-oriented interventions [46,47]. However, acceptance and mindfulness should not be used as interchangeable terms. Some studies have revealed that when a facet of mindfulness (i.e., observe the present moment experience) is applied without acceptance, it does not reduce negative emotional reactions [48,49]. Similarly, Teper & Inzlicht [50] suggested that mindfulness may dampen emotional reactivity to all sorts of external stimuli and, specifically, that the acceptance facet of mindfulness is mainly responsible for this dampening. Because this review focuses on the acceptance facet, studies that incorporated mindfulness-based instructions, but without specifying that acceptance was used, were excluded from this systematic review.

To be included in the systematic review, studies had to target children, adolescents, adults, and the elderly in clinical and non-clinical samples. Only studies with psychophysiological measures and experimentally induced pain were included. Pain induction was considered in any of the following stimulation modalities: electrical stimuli, mechanical stimuli, or thermal stimuli (see [51]). Clinical studies where pain was not induced (i.e., studies with patients who regulate their endogenous pain) were excluded. Furthermore, reviews, meta-analyses, dissertations, study protocols, and conference abstracts were also excluded. Finally, only studies published in English or Spanish were included, with no restrictions based on the year of publication.

### Identification and selection of studies

The screening, identification, and selection process was conducted by two independent reviewers (IJ and AD-G). First, studies were screened by reading titles and abstracts to identify potentially relevant articles, and those that were clearly ineligible were rejected. In the second phase, the reviewers independently assessed full-text versions of the relevant articles to determine final eligibility. In addition, the reviewers categorized the studies independently according to the ER instructions used (i.e., cognitive reappraisal or acceptance). The label used in the study was prioritized in the categorization. If a study did not use one of the specific terms used in the search strategy, such as cognitive reappraisal or acceptance, each reviewer classified the instruction independently on the basis of the definitions of cognitive reappraisal and acceptance proposed by Gross [52] and Hayes [23], respectively. After this classification by the authors, agreement was checked. In all the cases, the reviewers agreed on the classification of the strategies. Finally, the final selection of the studies to be included was supervised by two expert evaluators (MP and AG-P).

### Data extraction and coding

Data about the included studies were extracted in a data extraction form. The following variables were included: a) study (authors and year of publication); b) population (clinical or non-clinical population); c) aims of the study; d) sample (sample size); e) age (mean age of the sample); f) percentage of females; g) design of the study; h) comparator; i) ER strategy used (including the instructions given); j) moment when the ER strategy is used; k) psychophysiological measures; l) moment when the psychophysiological measures are assessed; m) type of pain induction; and n) main findings. All the variables mentioned above were extracted and coded independently by IJ and AD-G, and disagreements were resolved through discussion with a third author (MP).

### Risk of bias within studies

Risk of bias was assessed for each s`tudy independently by two team members (IJ, AD-G) using the Methodological Index for Non-Randomized Studies (MINORS) [53]. In this review, ten types of biases were evaluated qualitatively: a clear stated aim, criteria for participant inclusion, prospective collection of data, endpoints appropriate for the aim of the study, unbiased assessment until the study endpoint, calculation of the study size, an adequate control group, contemporary groups, baseline equivalence of groups, and adequate statistical analysis. Biases involving follow-up and the percentage of participants lost to follow-up were not assessed in this review because they were not applicable.

Studies were scored on an individual bias by indicating 0 (not reported), 1 (reported but inadequate), or 2 (reported and adequate). Conflicts about ratings were resolved through discussion and consensus.

## Results

### Selection and inclusion of studies

**Fig 1:**
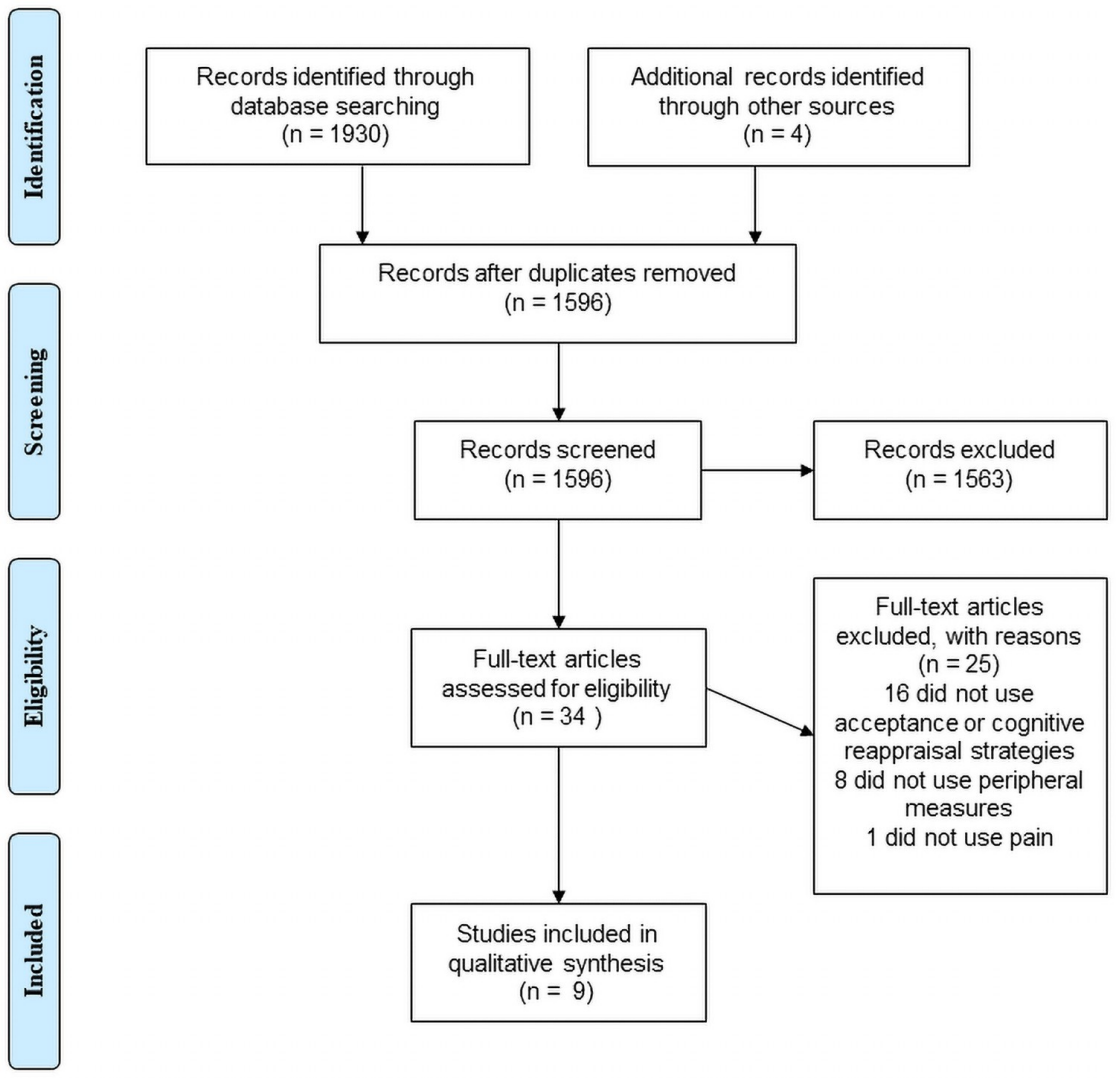
PRISMA flow diagram.

The search in the four electronic databases generated a total of 1930 potential studies (PubMed = 594; Web of Science = 598; PsycINFO = 238; Cochrane Library = 500). In addition, four studies were obtained through other sources (i.e., Google Scholar and references from relevant articles). Therefore, 1934 records were identified. After retrieving duplicates (n = 338), a total of 1596 studies were screened by two independent researchers (IJ, AD-G), based on titles and abstracts (S1 File). Of them, 34 full-text versions were assessed for eligibility, providing the reasons for exclusion: a) no use of cognitive reappraisal or acceptance strategies (n = 16); b) no use of induced pain (n = 1); and c) no inclusion of peripheral psychophysiological measures (n = 8). Thus, nine studies were selected for final inclusion in this systematic review. The study selection process is presented in the PRISMA flowchart (Fig 1).

### Characteristics of included studies

Relevant characteristics of the included studies are shown in Table 1. The research included in this systematic review consisted of experimental studies designed to test whether ER strategies (i.e., acceptance and cognitive reappraisal) influence the pain experience when using experimental tasks with induced pain. All the studies assessed non-clinical populations (i.e., college students, undergraduate university students, members of the general public, community members, and healthy populations). The mean age of the study samples ranged between 18.98 and 27 years, and the majority of the studies had a percentage of female participants, with only one study being conducted entirely with a male-only population.

**Table 1:** Characteristics of included studies.

Study	Sample size (N)	Age, M (SD)	% Female	Diagnosis (clinical or non-clinical population)	Aim	Design	ER strategy instructions	Comparator/ Control instruction	Strategy utilization period	Measures	Type of pain stimulation	Main findings
Braams et al., [60]	123	21.7 (5.1)	46.3	Non-clinical population (undergraduates and members of the general public)	To compare differences in pain, anxiety, and associated physiology (i.e., heart rate) between groups using suppression versus acceptance regulation strategies, or a no regulation strategy (i.e., control group) in response to experimentally induced pain	Within and between groups	Acceptance	Control: no instructions	Anticipation and shock phases	Pain and anxiety ratings Heart rate responses	Electric shocks S: right wrist D: 200ms	Acceptance led to comparable reductions in pain reports and cardiac defense responses, compared to the control condition, as well as greater reductions in reports of anticipatory anxiety compared to suppression.
Denson et al., [54] Study 2	90	21.57 (4.20)	57.77	Non-clinical population (undergraduates and members of the general public)	To test the hypothesis that reappraisal would lower psychological threat perceptions and enhance feelings of challenge, self-efficacy, and control over the stressor.	Between groups	Cognitive reappraisal	Control group: no instructions	Anticipation period (to mentally prepare for the cold pressor task over the next 10 min.)	Pain ratings Affect ratings Heart Rate	Cold pressor S: non-dominant hand T: 7 ± 1°C D: as long as possible, but no longer than 2 min	Participants in the cognitive reappraisal group reported enhanced anticipatory psychological appraisals of self-efficacy and control and greater post-stressor self-efficacy. Heart Rate effects were not found.
Evans et al., [61]	63	18.98 (1.62)	46	Non-clinical population (undergraduates)	To test two hypotheses regarding the relationships between unfamiliar mindfulness strategies (observe, describe, and accept), HRV, and pain tolerance.	Between-groups	Acceptance	Control group: instructions to respond naturally	During the cold pressure task	Pain tolerance Heart Rate variability	Cold pressor S: hand T: 0.27°C D: as long as possible	Mindfulness groups (*observe-only* and *observe*, *describe*, *and accept*) showed significantly less pain tolerance compared to the control group Higher Heart Rate Variability predicted greater pain tolerance, only in the control group.
Kalisch et al., [58]	18	27 (7)	55.56	Non-clinical population	To test whether the strategy of detachment attenuates subjective and physiological measures of anticipatory anxiety for pain	Within-groups	Reappraisal	Control group: Actively focus on the emotion	Anticipation	Subjective anxiety Heart rate Skin conductance	Electric shock S: back of the left or right hand (balanced between subjects) D: 1 sec	Subjective anxiety, heart rate, and skin conductance levels were lower when participants had to regulate their emotions through reappraisal strategy, compared to the control condition.
Hampton et al., [55]	142	20.78 (3.20)	68	Non-clinical population (undergraduates)	To examine the effects of emotion suppression and cognitive reappraisal on automatic (i.e., nonverbal) and cognitively mediated (i.e., verbal) pain expressions.	Between-groups	Cognitive reappraisal	Control: instructions to respond naturally	During the painful task	Pain ratings Anxiety and tension scales Heart rate Galvanic skin responses	Thermal pain stimulator S: The volar side of the left forearm, approximately 15 cm above the wrist T: 32 °C to 47 °C D: 5 seconds at 47°C, with a 7°C per second ramp-up and ramp-down	Reappraisal induction led to reductions in nonverbal and cognitively mediated (e.g., verbal) and automatic (e.g., facial activity) expressions of pain. However, effects of reappraisal were not found for heart rate and galvanic skin response.
Haspert et al., [62]	30	25.37, (3.58)	53.33	Non-clinical population (undergraduates)	To test the successful reduction of experimentally induced pain through acceptance-based regulation.	Within-subjects	Acceptance	Control: instructions to not regulate	From the 5 sec. before the thermal stimulation to the end of the trial (20 sec)	Pain ratings Heart Rate Skin conductance	Thermal pain stimulation S: The volar forearm of the non-dominant hand T: A level of thermal sensation that went from hot to just painful (from 35 to 49°C plus 1°C) D: 10 s	Acceptance was associated with lower pain intensity and unpleasantness. Heart rate was significantly lower during acceptance compared to control trials, whereas skin conductance revealed no significant differences.
Holmes & Houston, [59]	64	Age not reported	50	Non-clinical population (undergraduates)	To examine the effectiveness of specific strategies (redefinition and affective isolation) for handling stress.	Between-groups	Reappraisal	Control: no instructions	Anticipation and pain period	Affective ratings Pulse Rate Skin resistance	Electric shock S: Arm	Reappraisal was effective in reducing self-reported stress and pulse rate, and in increasing skin resistance. Reappraisal was not effective in reducing the pulse rate during the anticipation period, but it was effective during the stimulation period. Skin resistance was effective during both periods.
Lapate et al., [56] Study 2	24	22(2.1)	0	Non-clinical population (undergraduates)	To examine whether a common self-regulatory ability impacts the experience of both emotion and pain	Within groups	Reappraisal	Control: instructions to respond naturally	From four seconds before the pain stimulus to the end of the thermal stimulation delivery	Pain unpleasant and intensity ratings Heart Rate Pupil diameter	Thermal pain S: Left forearm T: A level of pain rated as “8 out of 10” (with a maximum of 49°C) D:18–12 sec	Reappraisal was associated with less pain unpleasantness and lower heart rate responses to the heat, compared to the enhanced condition. Reappraisal was associated with smaller pupil size and less corrugator activity, compared to the maintain condition. Unpleasantness ratings were positively correlated with heart rate changes.
Matthewson et al., [57] Study 1	41	24,3 (5.6)	48.78	Non-clinical population	To examine whether self-regulation influences pain-related physiology by developing pain predictive physiological markers based on skin conductance responses and electrocardiogram data	Within-groups	Reappraisal	Control: instruction to not regulate	During the thermal stimulation	Electrocardiogram Skin conductance responses Generalized Labeled Magnitude Scale	Thermal pain S: Three sites located on the middle forearm that alternated between runs. T: Three levels of temperature (level 1: 44.3°C; level 2: 45.3°C; level 3: 46.3°C; level 4: 47.3°C; level 5: 48.3°C; and level 6: 49.3°C). D: 12.5 sec seconds, with 3-second ramp-up and 2-second ramp- down periods and 7.5 seconds at target temperature.	Reappraisal produced decreases in intensity and unpleasantness ratings of pain, and it marginally decreased electrocardiogram and skin conductance responses. Electrocardiogram and skin conductance responses predicted self-reports.

S: Site where pain stimulation was induced; T: Temperature; D: Pain duration.

Regarding the ER strategy used, six studies included reappraisal [54–59], and three included acceptance [60–62]. All the studies compared reappraisal or acceptance to control groups/conditions or other regulatory strategies (i.e., suppression, enhancing negative emotions). No study compared these two strategies with each other.

Of the nine studies included in this review, six focused in the regulation of pain by including at least one of the following subjective ratings of pain: intensity, unpleasantness, tolerance, and threshold [54–57,61,62]. Moreover, some of these studies complemented these measures with affective ratings [54,55]. Nevertheless, three studies focused mainly on the regulation of anticipatory anxiety related to upcoming pain, with two of these studies including only anxiety reports [58,59] and one of them including both anxiety and pain reports [60].

Regarding psychophysiology, all the studies in this review included cardiovascular measures. Specifically, six of the studies included Heart Rate [54–56,58,60,62], one included Pulse Rate [59], one measured Heart Rate Variability [61], and one included the average of the Inter-Beat Interval [57]. In addition to cardiovascular measures, five studies reported skin conductance responses [55,57–59,62], and only one study also included pupil diameter and corrugator activity [56].

Finally, in connection with the type of pain induction used in the experimental task, two of the studies used the cold pressor task [54,61], four used thermal stimulation [55–57,62], and three used electric shock [58–60]. The studies conducted with cold pressor used between-group designs where the comparator was a control group, whereas the studies conducted with thermal and electric stimuli used between- and/or within-group designs in which the comparator might also be an unregulated block or a monitoring control condition.

### Emotion regulation instructions

Of the studies that used reappraisal strategies, two included instructions for reinterpreting the emotional stimulus as a good outcome, specifically instructing participants to “imagine the pain from a hot tub” [56] or to “minimize danger and enhance the counterfactual pleasantness of the stimulation” [57]. Another study used a change of perspective, taking a detached observer position as an ER strategy [58]. In this study, they encouraged the participants to distance themselves from their unpleasant feelings and thoughts. Finally, three studies used a combination of reappraisal regulation strategies [54,55,59]. First, Denson et al. [54] provided participants with different instructions based on reappraising emotional responses (e.g., “adopt a neutral and objective attitude toward their performance”) and changing their perspective (e.g., “think about it from a third-person perspective”). Second, participants in the study by Hampton et al. [55] were given instructions to reappraise their emotional responses (e.g., “change your thoughts, and the way you are thinking about your behaviors like facial expressions, and physical reactions like heart rate, in such a way that you don’t feel any discomfort at all”), change the stimulus (e.g., “feeling the warmth of the sun on his or her skin”), and change the perspective (e.g., “others may try to think of this experience as an opportunity to learn about psychological experiments rather than as a painful event”). Finally, Holmes & Houston [59] used an instruction based on reappraising the emotional stimuli (e.g., to think about the shock as a “vibrating sensation”) or change in perspective (e.g., “have a completely detached attitude toward the shock”) strategies.

Regarding acceptance strategies, Braams et al. [60] used brief instructions to “fully experience and accept any feelings and responses…without trying to control, avoid, resist or change them”. Haspert et al. [62] used an acceptance strategy based on Acceptance and Commitment Therapy, focusing on acceptance, mindfulness, and cognitive diffusion processes. Specifically, participants were instructed to accept their feelings, allowing any experience to occur without evaluating it. Furthermore, participants might employ the metaphor of the “cloud in the sky” [63] as a method of detachment. Evans et al. [61] taught participants acceptance strategies based on mindfulness-based stress reduction programs emphasizing a non-judgmental attitude [64].

### Control instructions

Emotion regulation studies have used a wide variety of control instructions to draw comparisons with emotion regulation conditions. For example, three studies did not give participants any instructions [54,59,60], whereas others instructed the participants to “perceive” and “sense the pain as it is and not use any strategies” [62] or “try not to regulate or change your sensation” [57]. Moreover, three studies used strategies such as “respond naturally” [55,56,61]. Finally, one study [58] used a control condition based on imagery and instructed participants to focus on the emotion and the way it affects their bodies and minds. It was similar to an emotion regulation condition (i.e., detachment) in terms of subvocal rehearsal, visual imagery, emotional awareness, and acceptance.

### The cognitive reappraisal strategy and self-reported and psychophysiological measures

Regarding self-reported measures, two studies found that cognitive reappraisal reduced the subjective emotional experience of the unpleasantness and intensity of pain [55,57]. In addition, one of these studies showed that reappraisal increased the pain threshold and tolerance level [55]. Another study reported that subjective anxiety produced by pain anticipation was lower when participants used the reappraisal strategy, compared to a control condition [58]. Furthermore, one study found that cognitive reappraisal was effective in reducing stress [59]. Finally, one study [54] showed that reappraisal increased feelings of control, challenge, and self-efficacy about the upcoming pain. Furthermore, participants in the reappraisal condition also felt that they had been more efficacious after the pain task than participants in the control condition.

With regard to the psychophysiological effects of cognitive reappraisal, all the studies included cardiovascular measures, four used electrodermal activity, and one included corrugator and pupil diameter. Diminished cardiovascular responses were found in three studies when participants had to reappraise [56,58,59]. In addition to these results, two studies did not find these differences [54,55], and one study found a marginally significant effect [57]. Holmes & Houston [59] found that reappraisal was effective in reducing cardiovascular responses during the stimulation period, but not during the anticipation period. In terms of electrodermal activity, two studies found lower responses when participants were using reappraisal in both the stimulation and anticipation periods, compared to a control condition [58,59]. Likewise, one study reported decreases in skin response activity when participants reappraised, compared to when they up-regulated their emotions, but these results were only marginally significant [57]. Additionally, this study found that electrodermal and cardiovascular responses across the trials predicted the unpleasantness and intensity of pain self-reports. In contrast, one study did not replicate any electrodermal activity effects [55]. Only one study included pupil diameter dilation [56], finding a smaller diameter size when participants had to reappraise, compared to the maintain condition. Moreover, this last study included corrugator electromyography, revealing that the use of reappraisal reduces corrugator activity, compared to the maintain condition.

### The acceptance strategy and self-reported and psychophysiological measures

With regard to self-reported measures, one study found that the strategy of acceptance was effective in reducing both pain and anxiety ratings [60]. Although this effect was also found for the suppression condition, acceptance was more effective in reducing anxiety produced by pain anticipation, compared to the control or suppression group. Likewise, another study reported lower pain ratings for acceptance, compared to the control condition [62]. In terms of tolerance, one study found that participants who received brief acceptance instructions had less tolerance to pain during the cold pressor task, compared to a control group that used familiar strategies to cope with pain [61], that is, any coping strategy that came naturally to them.

In terms of psychophysiology, all the studies included cardiovascular measures (i.e., two used Heart Rate, and one used Heart Rate Variability), and one included skin conductance responses. Specifically for cardiovascular measures, the studies found heart rate reductions when the acceptance strategy was used [60,62]. Braams et al. [60] found that acceptance led to reductions in heart rate responses once the shock was delivered (8s following), compared to a control condition. However, no cardiovascular effects of using acceptance were found in previous phases (i.e., preparation and anticipation phases). Furthermore, Evans et al. [61] found that higher Heart Rate Variability predicted greater pain tolerance, but only in the control group that used familiar strategies to manage pain, whereas no correlation was found for the group instructed to *observe*, *describe*, *and accept*. According to the authors, these results suggest that unfamiliarity with using acceptance strategies while attempting to tolerate pain may shape self-regulatory strength. Regarding skin conductance, no effect was found when acceptance was used [62].

### Assessment of risk of bias

Table 2 summarizes the different biases related to the methodological quality of the studies included in this review, according to the MINORS [53]. All the studies reported a clear and adequate aim, as well as appropriate endpoints in accordance with these aims. Moreover, all the studies had an adequate control group managed in the same period as the experimental group (i.e., contemporary groups). Furthermore, most of the studies reported information about the inclusion criteria, and they performed adequate statistical analyses.

**Table 2:** Assessment of risk of bias.

	Aim	Inclusion	Prospective	Endpoints	Unbiased	Follow-up	Lost-follow-up	Size	Control	Contemporary	Baseline	Statistical analysis
**Braams et al**. [60]	2	0	0	2	0	NA	NA	0	2	2	0	2
**Denson et al**. [54]	2	2	0	2	2	NA	NA	0	2	2	2	2
**Evans et al**. [61]	2	2	0	2	0	NA	NA	0	2	2	2	2
**Kalisch et al**. [58]	2	2	0	2	0	NA	NA	0	2	2	0	2
**Hampton et al**. [55]	2	2	0	2	2	NA	NA	0	2	2	2	2
**Haspert et al**. [62]	2	2	0	2	0	NA	NA	2	2	2	0	2
**Holmes & Houston**, [59]	2	0	0	2	0	NA	NA	0	2	2	0	1
**Lapate et al**. [56]	2	2	0	2	0	NA	NA	0	2	2	0	2
**Mathewson, et al**. [57]	2	2	0	2	0	NA	NA	2	2	2	0	2

Note: Not reported = 0, reported but inadequate = 1, reported and adequate = 2, not applicable = NA.

Additionally, only two studies reported information about the calculations of the study size. Regarding the unbiased assessment of the study endpoint, only two of the nine studies reported blind evaluation of objective and/or subjective outcomes. With regard to the baseline equivalence of the groups, three of the nine studies reported on the similarity of the groups. Finally, none of the studies provided information about a protocol established before the beginning of the study.

## Discussion

In recent years, an increasing number of studies have focused on psychophysiological measures in order to shed light on the ER process (see [29]). However, to date, there are no systematic reviews that summarize the studies that include experimental tasks to assess the relationship between psychophysiological activity and ER strategies to manage pain. The study of peripheral psychophysiological responses is particularly useful in various pathologies because it allows us to obtain objective measures of psychological processes in a non-invasive way. For this reason, this systematic review aimed to explore the relationship between ER strategies (i.e., cognitive reappraisal and acceptance) and psychophysiological peripheral correlates in laboratory studies where pain was induced.

Our findings suggest that both cognitive reappraisal and acceptance strategies are effective in reducing negative pain-related self-reports, such as anxiety, unpleasantness, and intensity of pain. These findings are in line with previous literature that has shown the effectiveness of these strategies in decreasing negative affect produced by unpleasant stimuli (e.g., pictures, films) [29,31]. Although self-reported measures show the efficacy of these ER strategies for managing induced pain, the psychophysiological effects of the use of these strategies are still unclear. In this regard, the results of this systematic review show that, overall, subjective ratings are modulated by the ER strategies, whereas the findings are not the same for the psychophysiology measures.

Literature indicates that successful emotion regulation is commonly related to a reduction in sympathetic activity [65]. In this line, some studies included in this systematic review have found that the use of cognitive reappraisal was associated with decreases in electrodermal responses and pupil diameter, which might reflect a reduction in the emotional arousal associated with sympathetic activity [66,67]. Likewise, cardiovascular and corrugator responses were also reduced when participants were instructed to reappraise. In this regard, cardiovascular responses (e.g., heart rate) have been associated with parasympathetic activation of the autonomic system, and they are sensitive to valence changes in relation to a negative stimulus [68]. Moreover, corrugator activity has been widely used as an index of affective responses, so that higher activity is associated with greater displeasure [69]. Therefore, the findings obtained in some studies included in this systematic review [56,58,59] might indicate that cognitive reappraisal leads to reductions in the affective valence produced by pain. However, other studies did not show this association; instead, they found that cognitive reappraisal leads to marginal or no effects on the autonomic measures (i.e., electrodermal activity and heart rate). Similarly, all the studies included in this review reported that the use of acceptance instructions for the management of pain was associated with reductions in heart rate responses, compared to control conditions. However, no effects of acceptance on electrodermal responses were found. These findings might suggest that acceptance would be effective strategies for reducing the unpleasantness of experiencing pain, but the influence of these strategies in modulating the activation level in response to the emotional experience is less clear.

Regarding the inconsistencies found in the different studies, several explanations can be identified. First, the ER instructions given in the studies included in this review differ greatly. For example, some studies used cognitive reappraisal as a strategy focused on the reinterpretation of negative aspects of the stimulus [57], whereas other studies refer to cognitive reappraisal when participants are instructed to take a detached perspective [58]. In addition, detachment is a method that is also used in the instructions for acceptance [62]. The lack of consistency in the operationalization of reappraisal and acceptance and the similarities in their instructions in the different experimental tasks make it difficult to determine whether the effects are produced by a cognitive change or an experiential change. As Hayes [70] claims, accepting implies making contact with the stimulus functions of events directly and automatically, without acting based on their derived verbal functions. However, some authors refer to acceptance as a type of reappraisal focused on revaluating the emotional response [30]. In the same way, whereas some studies use long periods for acceptance training, allowing an experiential change, other studies use briefer acceptance instructions that are sometimes combined with a cognitive diffusion process. This process has some similarities with those commonly used as cognitive restructuring, such as taking a detached perspective. Divergences in ER instructions could also imply differences in autonomic, cognitive, and brain recruitment that may be reflected in different pain processing and psychophysiological results [27,30]. For this reason, it would be advisable to improve the conceptualization of these two ER strategies in order to draw firmer conclusions about the differential effects of the specific processes used in these studies. Furthermore, we encourage future researchers to specify the components of interest in the study design phase. For example, if mindfulness instructions are given, it would be advisable to report which specific facets the participants are supposed to implement during the task.

Additionally, relevant methodological differences have been found in the control conditions between studies. Whereas some studies did not give any instructions, other researchers instructed participants to respond to the pain as they normally would, or they gave them instructions that might be similar to mindfulness approaches where participants should observe and not judge their emotions. Previous literature reported that different control instructions can result in differences in self-reports and physiological activation [71]. Moreover, a meta-analysis conducted by Zaehringer et al. [25] revealed that the effects on electrodermal and cardiovascular measures were significant when the instruction to “view” was given, but null effects were found when the instruction was “respond naturally”. In this regard, some studies included in this review [55,61] did not find significant physiological differences between the reappraisal and control conditions when participants were instructed to “respond naturally”. We argue that, in the control condition, participants could be using another adaptive ER strategy or the strategies they are used to, thus being more flexible in their ER. These issues might also provide a plausible explanation for the fact that some studies were able to find significant differences between strategies such as reappraisal and suppression (considered adaptive and maladaptive strategies, respectively), but they were not able to find differences between adaptive strategies compared to control conditions [52]. Therefore, we conclude that the use of a control instruction telling participants to focus on the stimulus without regulating or trying to change their emotions might be a better comparator of an emotion regulation condition than “respond naturally”. Future research should study plausible divergences in the different control conditions commonly used in emotion regulation tasks.

A second possible reason for the inconsistencies found is that the individual differences in the ER style may influence self-regulatory efficacy during the experimental task [72,73]. Evans et al. [61] suggested that unfamiliarity with using acceptance strategies while attempting to tolerate pain may shape the self-regulatory strength and produce less tolerance to it. However, Hampton et al. [55] did not find a relationship between self-reported reappraisal tendencies and the pain threshold and tolerance. Future studies should provide a more detailed description of the participants’ familiarity with the ER strategy to better understand the results obtained.

Third, the type of design could have an impact on the effect size of the results because within designs imply that the participant changes the strategy during the task [30]. Participants might guess that the researcher is comparing different conditions, and results could be biased by effort, attention, or expectation processes. For this reason, it is important to make an additional effort in this direction, and future studies are needed to optimize the designs for studying the effects of ER and better understanding which cognitive processes are modulating these effects.

Finally, another reason for the inconsistencies found in the studies included in this review could be the type of task and pain stimulation used. In this regard, different methodologies were used. For example, reappraisal has been demonstrated to be less effective than other strategies such as distraction in intense emotional situations [74]. Furthermore, Matthewson et al., [57] revealed that self-regulation effects on autonomic measures are stronger as the unpleasantness and intensity of the stimulus increases. Therefore, future studies should include characteristics of the stimulus (e.g., type of stimulation, temperature, intensity, or unpleasantness) as moderators of ER success. Additionally, it is important to determine the specific moment when the ER strategy starts to be implemented because studies have shown different psychophysiological results in anticipatory and pain periods [59,60].

Previous research using self-report measures has shown the superiority of acceptance over cognitive restructuring for increasing tolerance to experimentally induced pain [25]. Nevertheless, in this systematic review, no study compared these two strategies, which highlights the lack of research on the emotion regulation and pain relationship using objective psychophysiological measures.

Regarding the assessment of risk of bias, the overall quality of the studies included in this systematic review was acceptable, specifically regarding the aim and the adequacy of the endpoints for the aim of the study. However, some studies did not properly report the inclusion criteria and sample size calculation. In addition, it is worth noting that no studies reported a protocol established before the beginning of the study. For this reason, we encourage authors to register study protocols that include methodological aspects, in order to improve the methodological quality of experimental studies. This would facilitate future replication of the studies and systematic reviews of the literature and reduce publication bias.

This work has some strengths and limitations. To our knowledge, this is the first review to systematically summarize the literature on the relationship between peripheral psychophysiology and two of the most widely used ER strategies (cognitive reappraisal and acceptance) in pain management. Moreover, this review conforms to PRISMA guidelines and has a previous record in PROSPERO. Nevertheless, our findings reveal a lack of studies in this field, which makes it difficult to draw clear conclusions about the effects of ER strategies on peripheral measures when participants are managing pain. In addition, it is possible that some studies were not located and have not been included in this review. Given that the review was conducted with three databases and there were language restrictions (English and Spanish), some studies might have been left out.

Furthermore, another limitation is related to the lack of consistency in the terms used for the strategies across the studies. For example, some studies used “suppress” or “down-regulate” when referring to reappraisal strategies [56,57]. Likewise, in numerous studies, the ER strategy used was open, not well-defined, or mixed with other strategies [75,76], and so these studies were excluded from this review. In addition, acceptance is a strategy that is often included in mindfulness programs, but all the studies that used mindfulness were excluded if they did not specify that a component of acceptance was included. Finally, our study only focuses on reappraisal and acceptance strategies, leaving out other ER strategies that may be of interest.

## Conclusions

The present review confirms that there are few studies focusing on psychophysiological activity and pain management through reappraisal and acceptance strategies. However, there is a growing interest in this topic.

Although cognitive reappraisal and acceptance strategies appear to be associated with decreased psychological responses, these findings are not found in all the studies. The inconsistencies found in this systematic review, in terms of ER concepts, instructions, and length of training in ER strategies, among other issues, indicate a lack of agreement about the procedures to follow in laboratory settings that can result in differences in physiological responses. Therefore, one important conclusion from this review is the need to advance toward a more standardized methodological framework in this line of research. Likewise, methodological factors, such as stimulus characteristics (e.g., type of pain, intensity) and the moment when the strategy is used, should be carefully explored to achieve a better understanding of the modulators that can underlie the effectiveness of ER strategies for pain.

In addition, further research is needed to determine the role of cognitive reappraisal and acceptance strategies in peripheral psychophysiological responses. Specifically, it would also be necessary to evaluate an aspect that was not considered in any of the psychophysiological studies included in this review, that is, comparing these two strategies and determining which one is more effective in managing pain. Importantly, new research should focus on comparing specific components or subtypes of both strategies (e.g., willingness, attention, taking a detached perspective), in order to determine the relationship of each cognitive process on the psychophysiological correlates.

## Supporting information

S1 Checklist PRISMA 2009 checklist. (DOC)

S1 File List of reviewed articles. (XLSX)
